# Observations on Hemeralopia, or Night Blindness

**Published:** 1816-09

**Authors:** H. Baillie

**Affiliations:** late Surgeon of His Majesty's Ship Ajax, in the Mediterranean


### *Observations on Hemeralopia, or Night Blindness.* By H. Baillie, late Surgeon of His Majesty's Ship Ajax, in the Mediterranean.

THE name, as well as the nature of this curious phænomenon, has given rise to discussions among the ancients and the moderns. I use the term Hemeralopia, from its most obvious derivation ἡμέρα, dies, and οπτω video, considering the L as added for the sake of euphony.

The Hemeralopia which appears among sailors in tropical and other warm climates, seems to differ, at least in its causes, from that described by medical men on shore. Scarpa, and most of the German writers, attribute the complaint to derangement of function in some of the chylo-poietic viscera, and trust principally to emetics, cathartics, and ultimately tonics for the cure. But among the class abovementioned, scorbutic diathesis and solar light or heat, appear the principal remote causes of night blindness; and in these cases the complaint is not to be removed by evacuations or tonics.

In most instances, Hemeralopia approaches gradually, till total nocturnal darkness takes place; and in protracted cases, there is a diurnal irritability of the retina and other coats of the eye, which renders a brilliant light very intolerable to the patient. Bontius asserts, that complete blindness has resulted from neglected, or long protracted Nyctalopia. Its duration is very various, especially when left to itself, as I have reason to think has pretty generally been the case formerly, from a total ignorance of its nature or treatment. Of the numerous absurd remedies which patients themselves have employed, as bullock's liver, shark oil, &c. I need not speak, since the narratives cannot be depended on, and they have totally failed in the hands of medical men.

Fifteen well marked cases have lately come under my care; and with respect to the reality of the disease, I had not the smallest doubt, from the well known good character of most of the men, who returned with alacrity to duty the moment their sight was restored.

The Hemeralopia appeared immediately after Lord Exmouth's first visit to Algiers, and was pretty generally ascribed by the patients themselves to a Siroc wind, which blew for a few hours, one forenoon, while the fleet lay off that town. It did not affect any particular constitution more than another; nor did the *colour* of the iris appear to give any predisposition to the individual, as it varied from a dark hazel to a light grey. With the exception of two, they were all young men, between nineteen and twenty-five years of age, who felt the influence of this complaint; and it was exclusively confined to the *topmen* and to the *marines* who did duty on the gangways, poop, and other exposed parts of the ship. Not a single case occurred among the gunner's crew, waisters, or after-guard; nor did any officer or petty officer suffer from this peculiar affection.

It is well known that the class of seamen denominated *topmen*, when not actually employed aloft, are very much in the habit of sleeping in the tops, exposed to the rays of the sun and to the influence of the weather; and in the Ajax, the marines, when not on sentinel duty, wore white jackets and trowsers with a cap of the same colour, which afforded no protection against the glare of light while on the gangways. Several of the men had experienced Hemeralopia on former occasions, either between the tropics, or in the Mediterranean; and one man in particular, had been afflicted for two years on the Jamaica station. The state of health did not appear at all affected, nor did the organ of vision itself afford any indication of disease. This, however, may be attributed to the celerity of the cure; for in prolonged states of the complaint, contractions of the pupil, and great irritability of the eye are pretty general attendants. During the absence of the sun the pupils here were somewhat dilated, and there was a certain degree of imperfect vision by strong candle-light.

Emetics, as recommended by Scarpa, were not employed by me; but I first tried repeated cathartics, with the external application of sulphas zinci in solution, from an idea that gastric or intestinal derangement might be the source of the visual affection. From this plan, not the smallest benefit was derived. I next exhibited the citric acid, in case any scorbutic diathesis lurked in the system; but this was equally as ineffective as the treatment by purgatives. At this time, a paper in the fifth volume of the Medico-Chirurgical Transactions, written by Mr. Bamfield, fell into my hands, and I instantly adopted the plan which experience had proved to him so successful, namely, the application of blisters to the temples. The effects of these were astonishing, and exceeded my most sanguine hopes. They acted like a charm; for in no one instance did the complaint resist this treatment more than three days; nor was a repetition of the blisters necessary in any case; the men returning regularly to their duty, as soon as the blistered surfaces were healed.

I consider that the profession at large, and the naval medical practitioner in particular, are much indebted to Mr. Bamfield for his excellent account of Hemeralopia, which does infinite credit to his talents as a close observer of morbid phænomena.*

I agree with this gentleman in the opinion that the remote cause of Hemeralopia is to be sought in too much light acting for a long period on the retina, and consequently that the proximate cause is probably an insuscep-

* This gentleman is now settled in general practice in the metropolis, where his talents will have ample scope, and where he will do honour to the profession. EDIT.

tibility to the stimulus of light, or a degree of paralysis in that delicate expansion of the optic nerve. It is certain that the disease prevails most where the solar beams are most vivid, and that Hyperboreans, who are unaccustomed to a tropical sun, are more frequently affected with the disease, than the indigenous inhabitants.

In respect to the treatment by blisters, I should conceive that Mr. Bamfield has the merit of priority, since the Hemeralopia which Sauvages affirms to have been once epidemic in some villages near Montpellier, and which gave way to *blisters*, emetics, cathartics, and other evacuations, appears to have been what is termed "*cæcitudo diurna*," or day blindness, a disease of a very different nature from the Nyctalopia of modern writers. But whether Mr. B. be the original proposer or not, he has certainly pointed out a very effectual mode of cure, as the cases abovementioned evince; and as the *corroboration* of a new method of treatment in an obstinate disease, is often of as much consequence as the *proposal* of it, I trust the foregoing facts and reflections may not be considered uninteresting, or undeserving a place in your valuable Journal.

Before closing this paper, permit me to observe, that *cynanche parotidea* appeared among the crew of his Majesty's ship Ajax, in the spring of 1815, and was evidently contagious; as the men who subsequently suffered from it, all belonged to the same mess, the same watch in the tops, or the same tier of hammock-berths, with those first affected.

In all of them there was considerable pyrexia; requiring the free use of the lancet, blisters, and every part of the antiphlogistic system. In only the three first cases were the testicles affected; and in two of *them* the attack of inflammation and swelling was synchronous in the salivary and seminal glands. In the third, it appeared like metastasis, since the inflammatory action subsided in the face as it increased in the testicles.

I am, &c.

H. BAILLIE.

London, July 20, 1816.

